# Insulin resistance with associated hyperinsulinemia as a risk factor for the development and worsening of HFpEF

**DOI:** 10.3389/fcvm.2026.1719492

**Published:** 2026-01-30

**Authors:** Serafino Fazio, Guido Carlomagno

**Affiliations:** 1 Federico II University School of Medicine, Naples, Italy; 2 Mediterranea Cardiocentro, Naples, Italy

**Keywords:** cardiovascular disease, concentric remodeling of left ventricle, diastolic heart failure, heart failure with normal ejection fraction, heart failure with preserved ejection fraction, hyperinsulinemia, insulin resistance, type 2 diabetes mellitus

## Abstract

The prevalence of insulin resistance (IR) with associated hyperinsulinemia (HI) is increasing worldwide, as is the prevalence of heart failure with preserved left ventricular ejection fraction (HFpEF). This narrative review aims to explore the epidemiological and pathophysiological relationship between IR/HI and HFpEF, the possible mechanisms by which IR/HI could underlie HFpEF development and worsening, and the actual and future therapeutic implications of this interplay. The prevalence of IR in patients with HF is not negligible, and we will go through the existing literature highlighting this epidemiological association and the longitudinal data supporting a causative link. We will give a brief overview of molecular and physiological mechanisms connecting IR and HFpEF, such as the alteration of vascular homeostasis resulting in endothelial dysfunction and arterial hypertension, myocardial and vascular wall cell growth resulting in microvascular and macrovascular alterations of coronary circulation, and concentric remodeling of the left ventricle resulting in increased stiffness and diastolic dysfunction. We will review the concept of “diabetic cardiomyopathy” as a study model of these correlations. Finally, we will go through existing antidiabetic drugs with a current or potential future role in the treatment of HFpEF and summarize evidence on lifestyle and rehabilitative interventions in the field. Many of the cardiovascular abnormalities caused by IR/HI may be a contributing factor to the development and worsening of HFpEF. Further research is warranted to explore whether early diagnosis and specific treatment of IR/HI in at-risk populations may prevent HFpEF or delay its progression.

## Introduction

The burden of insulin resistance (IR) with associated hyperinsulinemia (HI) is progressively increasing worldwide, both in industrialized and in economically emerging countries, reaching in some reports a prevalence up to 50% of the general population, primarily as a consequence of continuous and massive changes in dietary habits and lifestyle in general over the past two centuries (1–3). At the same time, along with the aging of the population and the reduced mortality of frequent communicable and chronic diseases, the prevalence of heart failure (HF) has been increasing particularly in elderly subjects, with approximately 50% of patients with HF characterized by a normal left ventricular ejection fraction (HFpEF) (4, 5).

**Figure 1:**
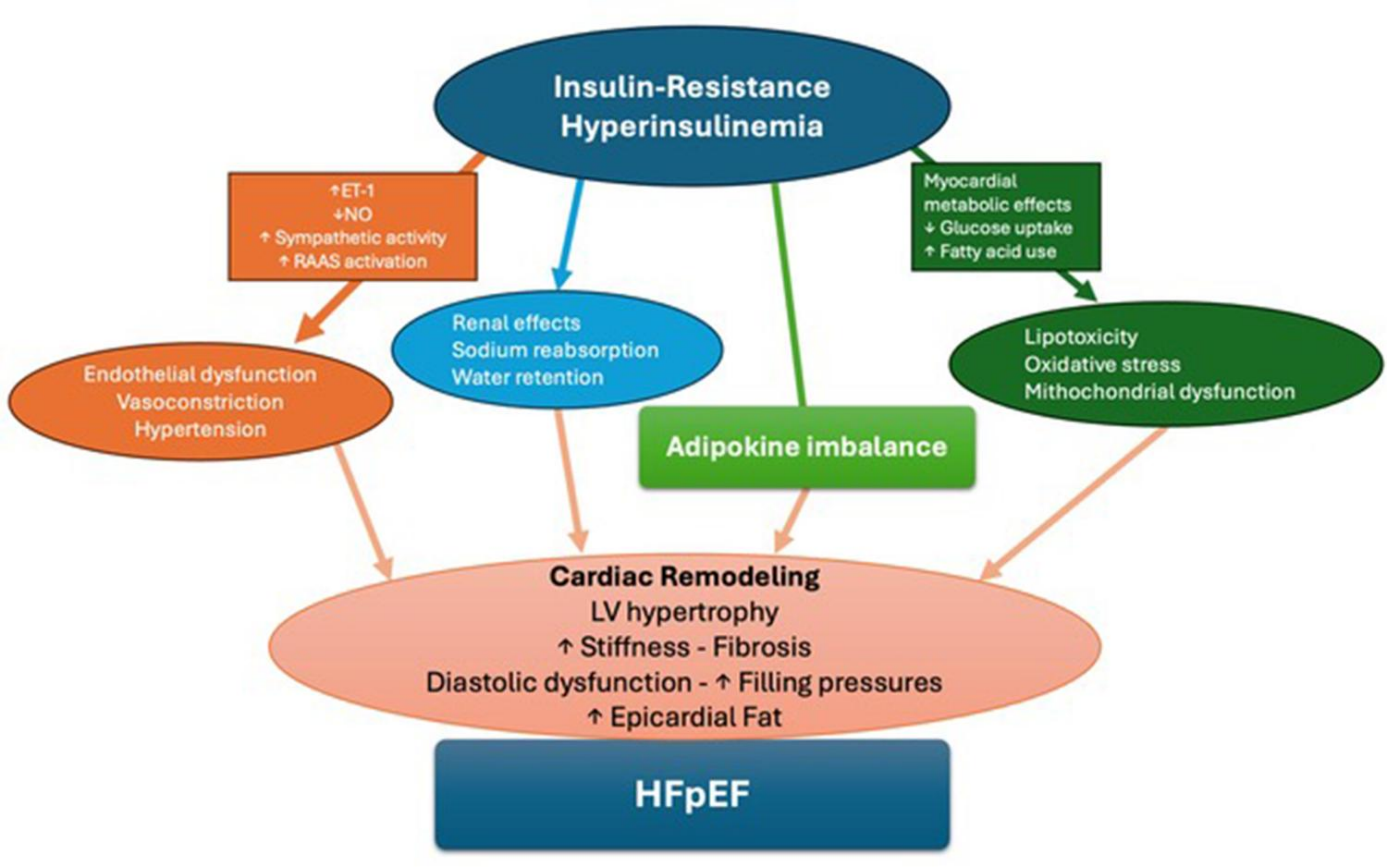
Central illustration. A summary of pathophysiological mechanisms connecting IR to HFpEF.

Epidemiological evidence suggests an association between IR and HF (6–9). This association seems to be supported by a sound pathophysiological rationale: literature shows that chronically increased levels of insulin associated with IR produce relevant abnormalities on the cardiovascular system, often long before the development of guideline-defined type 2 diabetes (T2DM) (10–14). These abnormalities might be an important contributing cause in the development and worsening of HFpEF. However, to date, the scientific community and healthcare institutions do not recognize IR as a risk factor for HFpEF, at least from a pragmatic standpoint (ie no indications exist to screen for and treat IR early).

This narrative review aims: 1. To describe the complex interplay between the cardiovascular abnormalities produced by IR/HI and the possibilities of development and worsening of HFpEF; 2. To hypothesize the potential practical consequences of treating IR/HI in the prevention and management of HFpEF; 3. To stimulate the scientific community and health institutions on this topic.

## Methods

For this narrative review, manuscripts dealing with the potential association between IR/HI and HFpEF have been searched in the major databases of scientific literature (PubMed, Science Direct, Scopus) from those published between 1990 and 2025, using the keywords: Insulin resistance, hyperinsulinemia, Heart Failure with preserved Ejection fraction, heart failure with normal ejection fraction, diastolic heart failure, type 2 diabetes mellitus, concentric remodeling of left ventricle, cardiovascular disease. Only relevant papers in English, performed with correct scientific design and published in peer-reviewed journals with good impact, were included. Case reports and congress abstracts were excluded, as were non-peer-reviewed papers. The manuscripts were revised independently by the two co-authors and the conclusions were shared and approved. Reference lists of selected articles were also analyzed, and occasional cited papers were added to the review using the same criteria.

## Definition and diagnosis of IR/HI

IR is characterized by the fact that a certain amount of insulin secreted by the pancreas has effects on glucose metabolism of a smaller magnitude than expected. To maintain blood glucose levels within the normal range, the pancreas is thus forced to secrete greater amounts of insulin. HI is, therefore, an emblematic and constant characteristic of insulin resistance. This, in the long run, induces a functional exhaustion of the Langerhans cells of the pancreas leading to the development of T2DM (14, 15). IR/HI precedes the development of T2DM, often by many years (14, 16), producing significant damage mainly, but not exclusively, at the cardiovascular level (17). Patients with newly diagnosed T2DM often already show cardiovascular complications, so much so that these subjects are, per guidelines, treated in secondary prevention (18, 19).

The gold standard for the diagnosis of insulin resistance is the hyperinsulinemic-euglycemic clamp, an invasive procedure unfit for screening purposes. Through the decades, many surrogate indices have shown good correlation with the clamp. Among these, the most frequently used are the Homeostatic Model Assessment (HOMA-IR) index, which takes into account fasting blood glucose and insulin, and the Triglyceride-Glucose (TyG) index, which is derived from fasting triglycerides and blood glucose [HOMA-IR = (fasting blood glucose mg/dL x fasting insulin µU/mL)/405; TyG = Ln(fasting triglycerides mg/dL x fasting blood glucose mg/dL/2)] (20–22).

## Pathophysiological links between IR and HFpEF

In subjects affected by IR there is generally a defect in the receptor and/or in some post-receptor pathways, such as that of phosphoinositide 3 kinase (PI3K), while other transduction pathways, for example that of the mitogen activated protein kinase (MAPK) stimulating cell differentiation and growth, are little or not at all altered, so that increased circulating levels of insulin end up hyperactivating them (10, 14, 16).

### Endothelial dysfunction

The homeostasis of the arterial circulation is regulated above all by a balance between the secretion of vasoconstrictor (e.g., endothelin-1, ET-1) and vasodilator substances (e.g., nitric oxide, NO). In conditions of IR this balance is profoundly altered in favor of ET-1. This is because there is a reduced secretion of NO, due to the alteration of the PI3K pathway, and, vice versa, an activation of the secretion of ET-1 due to the increased signaling of the MAPK pathway. This produces vasoconstriction, altered district flows and endothelial dysfunction, triggering and/or worsening the atherosclerotic process. In addition, increased circulating insulin, also binding to insulin like growth factor-1 (IGF-1) receptors and increasing MAPK pathway signaling, stimulates the proliferation of vascular smooth muscle cells and endothelial cells, producing thickening and stiffening of the vascular wall and further fostering atherosclerosis (23, 24).

### Myocardial remodeling

HI is also characterized by an increase in the activity of the sympathetic nervous system. Euglycemic clamp experiments with increasing doses of insulin show that higher circulating insulin levels determine significant increases in the levels of circulating norepinephrine (25, 26). A hyperactivation of the renin-angiotensin-aldosterone system (RAAS) has also been described in states of HI, with potential influence on vascular homeostasis and cardiac fibrosis (27).

It is also known that insulin binding to renal tubular receptors causes sodium reabsorption and water retention. The combination of all the above-mentioned mechanisms (vasoconstriction due to the prevalent action of ET-1, vasoconstriction due to excess circulating norepinephrine, activation of the RAAS and increased sodium and water reabsorption) will facilitate arterial hypertension (28).

High blood pressure, along with the direct stimulus to the growth of myocardial cells and with the coronary micro- and macro-circulatory alterations, determines concentric remodeling of the left ventricle with increased stiffness and diastolic dysfunction (29, 30). This is also contributed to by the accumulation of perivisceral fat around the heart and between the myocardial fibers associated with IR (31–33) (Figure 1).

### Metabolic behavior of myocardium

IR/HI also influence the intrinsic metabolic behavior of the myocardium. Normally, the myocardium can employ both glucose and fatty acids as substrates, in a state of metabolic flexibility. Stress states should lead to a substrate shift towards glucose to increase energy efficiency. IR prevents this adaptive response and may cause further injury by reducing glucose uptake and increasing fatty acid delivery, contributing to lipotoxicity, inflammation, oxidative stress, and fibrosis. Well-characterized animal models of IR cardiomyopathy demonstrate inefficient energy metabolism (34).

### The adipokine hypothesis

Recently, a “grand unifying theory” of cardiometabolic disruption has been proposed: in this framework, the development and progression of HFpEF is promoted by an imbalance in the secretion of “adipokines”, a heterogeneous group of endocrine and paracrine molecules acting on the heart and vessels, whose production and effects are directly or indirectly regulated by adipose tissue; patients with HFpEF usually have central adiposity and display enhanced secretion of cardiotoxic adipokines, along with defective production of cardioprotective ones (35). This theory, to some extent, “includes” IR/HI as one of the mediating pathways of the dysregulation.

## Epidemiology: IR as risk factor for HFpEF

HFpEF, previously called “diastolic” heart failure, is currently defined as a heart failure syndrome with a left ventricular EF ≥ 50% and evidence of spontaneously or provocatively increased LV filling pressures (36); according to this definition and in contemporary populations, HFpEF comprises approximately 50% of all HF cases (37). The signs and symptoms of HFpEF are similar to those of other HF subtypes, but the pathophysiological processes and determining risk factors may differ. These differences make the diagnosis of HFpEF relatively challenging. Patients with HFpEF have increased all-cause mortality, reduced quality of life, and significantly increased social and health care costs (15).

IR is a frequent condition both in western countries and in economically emerging areas, with a global estimated prevalence of ∼26% in the general adult population and approaching 40%–50% in some geographical areas (38). Longitudinal data show that its prevalence has been increasing in recent years across most regions and age groups, a surge most probably caused by dietary and lifestyle changes leading to overweight and obesity, especially in countries experiencing a rapid economic growth (39–41). Large-scale databases demonstrate that elevated surrogate indices of IR are associated with cardiovascular events and increased all-cause mortality during long-term follow-up, suggesting that the presence and extent of IR may have a prognostic impact in non-diabetic subjects as well as in patients who already meet criteria for T2DM (42, 43).

Overweight, obesity, and age are important confounding factors in interpreting associative data between IR/HI and both increased cardiovascular risk and HF development (44, 45). However, it is known that both overweight and obesity and aging are conditions of IR and that this is widely present in women from menopause onwards (46–48).

Clinical studies in humans strongly support the existence of a link between IR and non-ischemic HF. Contemporary cohorts report a prevalence of central adiposity and IR exceeding 50%, with and without T2DM, in subjects with HFpEF (49). As a matter of fact, IR seems to be more prevalent in HF patients even with a normal body weight (50).

Conversely, in large at-risk populations, the presence of IR strongly correlates with a diagnosis of HFpEF (51). Longitudinal studies also support the notion that IR itself is a strong risk factor for future development of heart failure, with some studies pointing at a more specific role in HFpEF vs. HFrEF (52, 53). These epidemiological associations may reflect a causal relationship between the two entities, although significant confounding factors certainly exist, as IR may simply be one of the hallmarks of the pathologic milieu underlying HFpEF.

Moreover, several studies have reported progressive pre-clinical changes in cardiac structure and function of IR subjects—such as LV hypertrophy, diastolic dysfunction and decreased longitudinal LV deformation—well before symptomatic HF has developed (54–58). These seem to be independent of common contributing factors, i.e., hypertension and body mass index, and appear to be more pronounced in women, especially with full-blown diabetes and at advanced age. This knowledge dates back several decades, as already in the nineties studies showed that glucose and insulin levels correlated with relative wall thickness independent of age, systolic blood pressure, and body mass index (56).

Some epidemiological evidence suggests a significant impact of IR in the prognosis of HFpEF patients. A recent study on a large Chinese HFpEF population reported a significant prognostic impact of the triglyceride-glucose index (TyG) beyond currently used risk scores (59).

A combined analysis of large HFpEF trials reports that insulin use—a surrogate marker of increased IR/HI but also of greater clinical complexity—is a marker of worse clinical outcomes and higher incidence of sudden cardiac death (60).

In summary, IR is prevalent in the non-ischemic HF population even in the absence of T2DM and obesity, often precedes and predicts the development of HF, and may represent an independent risk factor for worse prognosis.

## Diabetic HF syndromes and diabetic cardiomyopathy

While most diabetic patients affected by HF show “common” cardiac pictures undistinguishable from those of non-diabetic subjects, the last two decades have seen mounting evidence supporting the existence of a true “diabetic cardiomyopathy” (DbCM). This is usually defined as a form of heart failure not sustained by inheritable causes, ischemia or valvular damage in a diabetic person displaying signs of intrinsic myocardial dysfunction. HI and IR are key drivers of DbCM, leading to concentric remodeling of the left ventricle, increased left ventricular mass, and diastolic dysfunction (55, 61–63).

The prevalence of DbCM seems non-negligible, with reported rates of 1%–3% of the entire T2DM population and 10%–20% of diabetic HF patients (64). Subjects without overt HF but with instrumental findings suggestive of DbCM—cardiac remodeling and elevated cardiac biomarkers—seem to represent up to >10% of the entire T2DM population and demonstrate high rates of progression to symptomatic HF (65).

DbCM may in fact resemble both main HF phenotypes: HFrEF—with dilative disease with severely to mildly reduced EF—and HFpEF—with cardiac hypertrophy, normal EF and restrictive diastolic physiology. These two appear as distinct phenotypes, and “progression” from HFpEF to a hypokinetic phenotype seems relatively rare in the absence of an acute ischemic event (66).

Besides the mechanisms induced by IR at myocardial and vascular levels (see paragraph above), several other pathophysiological pathways have been advocated as initiators and promoters of DbCM. Some are worth mentioning, i.e., microvascular dysfunction leading to tissue ischemia with patent epicardial coronary arteries, diabetes-associated autonomic neuropathy, accumulation of advance glycation end-products (AGEs), oxidative stress both on the mitochondria and the endoplasmic reticulum (67). Trials of metabolic modulators are underway as a candidate specific treatment of DbCM (ARISE-HF trial) (68).

## Diabetes drugs in HFpEF

Published literature suggests that most of the drugs for the treatment of T2DM which act by reducing IR and HI may also improve outcomes in diabetic HFpEF patients. Among these, there is one known and used for many years, namely Metformin, and two newer classes of drugs, namely sodium-glucose cotransporter 2 inhibitors (SGLT2 Is) and glucagon-like peptide 1 receptor agonists (GLP1-RAs). In addition, there is also a natural substance, used for many years as an anti-diabetic in Eastern cultures, namely berberine.

### Metformin

Metformin is a biguanide used orally as a treatment to improve insulin sensitivity in IR conditions such as diabetes, prediabetes, and polycystic ovary syndrome. The increased peripheral glucose utilization following metformin treatment most likely results from the induction of glucose transporter 4 (GLUT4) expression and its increased translocation to the cytoplasmic membrane of target cells. However, the mechanisms underlying the insulin-sensitizing effects of metformin are not yet fully defined (69).

Metformin treatment has been suggested to improve diastolic function in patients with T2DM; in an echo study, the use of metformin was associated with a shorter mean isovolumic relaxation time (IVRT) and higher e′ values, independent of concomitant use of sulfonylureas or insulin (70). In the MET-REMODEL study using cardiac magnetic resonance in non-diabetic patients with ischemic heart disease and IR, metformin treatment significantly reduced left ventricular hypertrophy compared with placebo (71). Multiple lines of experimental *in vivo* and *in vitro* evidence give mechanistic insights into these clinical data, with documented effects on myocardial metabolism, vascular function, and insulin-resistance (72).

Classic data from UKPDS already highlighted that metformin improved clinical outcomes in obese T2DM patients, even on top of insulin treatment, although the number of patients allocated to treatment with metformin was less than 10% of all those randomized. Conversely, insulin and sulfonylureas were equally detrimental in obese people, probably due to worsening HI. This probably indicates that to obtain an improvement in outcomes it is necessary to act with drugs that reduce IR and therefore the levels of circulating insulin (73). This is confirmed by more recent registry data: initiation of treatment with metformin in patients with T2DM and HF was independently associated with reduced risk of mortality and HF hospitalizations, while initiation of sulfonylureas worsened outcomes (74).

Despite a traditional contraindication in HF due to concerns regarding the risk of lactic acidosis in the setting of tissue hypoperfusion, many diabetic HF patients are therefore treated with metformin based on clinical experience and more recent evidence (75). A recent meta-analysis suggests that treatment with metformin may provide a mortality benefit in diabetic patients with HFpEF (76). Prospective trials are underway to explore the impact of metformin on contemporary HF cohorts (DANHEART) and on cardiovascular outcomes in non-diabetic subjects (VA-IMPACT trial).

### SGLT2-Is

SGLT2-Is (mainly Empagliflozin and Dapagliflozin) have been increasingly used in treatment of T2DM since their approval about 10 years ago. These drugs determine a reduction in blood glucose due to increased urinary excretion by blocking the sodium-glucose cotransporter 2 in the proximal tubule of the kidneys. Circulating insulin levels needed to maintain blood glucose are thus reduced, with improved IR indices and probably less damage from HI (77). It has been shown that SGLT2-Is play a role in reducing LV mass, reversing adverse cardiac remodeling and improving LV systolic function in HF patients (78). Empagliflozin given to patients with T2DM and a history of cardiovascular disease resulted in a reduction in LVM and end-diastolic volume, which were associated with an improvement in LV diastolic function parameters already after 3 to 6 months of treatment; these data were consistent both by Doppler echocardiography and MRI (79–82).

SGLT2-Is significantly reduce mortality and cardiovascular hospitalizations in patients with HF (83). The mechanisms by which this occurs in HFpEF are not yet fully understood. Chronic SGLT2-I-induced natriuresis and osmotic diuresis may exert beneficial effects on blood pressure and hypervolemia in HF. These agents have been shown to reduce epicardial adipose tissue and alter adipokine signaling, which may play an important role in the reduction of inflammation and oxidative stress observed with SGLT2-Is (35). Finally, SGLT2-Is have been shown to reduce myofilament stiffness as well as extracellular matrix remodeling/fibrosis in the heart, improving diastolic function (84).

A recent comprehensive meta-analysis underlines that, in addition to known benefits on mortality and hospitalizations, SGLT2-Is treatment is associated with significant improvement in patient-relevant outcomes such as exercise capacity and quality of life measures, regardless of sex or ejection fraction (85).

SGLT2-Is are now recommended for the treatment of symptomatic HF independent of the presence of diabetes and across the entire spectrum of left ventricular ejection fraction (36).

### GLP1-RAs

GLP1-RAs have been marketed since the late 2000s for T2DM, and, later, also for the treatment of severe obesity. GLP1-RAs mimic the action of the hormone GLP-1, helping to control blood sugar and promote weight loss. GLP1-RAs act not only by reducing calorie intake and body weight, but also affecting the mechanisms involved in IR, i.e., increasing expression of glucose transporters in insulin-dependent tissues, decreasing inflammation, reducing oxidative stress, and modulating lipid metabolism (86, 87). Experimental evidence suggests that GLP1-RAs also exert immediate beneficial effects on insulin sensitivity independent of weight loss (88).

Trial evidence points at a reduction of major adverse cardiovascular events in patients treated with GLP1-RAs, and these are currently regarded as drugs of choice in patients who already experienced, or are at high risk for, macrovascular complications of T2DM (89). However, their efficacy on more HF-specific outcomes has less homogeneous evidence, depending on the molecule used and on trial design. Early trials using Liraglutide in HFrEF patients (FIGHT and LIVE trials) did not demonstrate any significant benefit in terms of LVEF, natriuretic peptides, and hard outcomes such as mortality and HF rehospitalizations, while negative safety signals due to increased arrhythmia and HF events have been suggested in the advanced HFrEF population of the FIGHT trial (90–92).

The strong pathophysiological rationale connecting T2DM, central adiposity, IR and HFpEF prompted, in the last few years, the initiation of specific trials. The STEP-HFpEF trial, a randomized placebo-controlled study using subcutaneous semaglutide in 529 non-diabetic obese patients with HFpEF, demonstrated a significant reduction in body weight and an improvement in quality of life measures after one year of treatment; a hierarchical secondary win-ratio analysis including HF events and exercise capacity (6 min walk) also suggested a beneficial effect of semaglutide (93). Similar results were basically replicated in T2DM patients with HFpEF in the STEP-HFpEF DM trial (94). Ancillary observations from these two studies suggest beneficial effects of the GLP1-RAs on systemic inflammation, cardiac remodeling and function, and loop diuretic use (95–97). Interestingly, the authors also showed that the benefit of Semaglutide was more pronounced in frail patients (98).

Tirzepatide is a combined GLP1/GIP (Glucose-dependent Insulinotropic Peptide) agonist, characterized by apparently superior efficacy on weight loss and indices of IR (99). The SUMMIT trial randomized 731 obese HFpEF patients to either Tirzepatide or placebo, demonstrating a significant reduction in worsening HF events in the treated arm after a median follow-up of two years (100). Secondary analyses resemble the results reported with Semaglutide, with significant effects on weight loss, walking distance, and inflammation. A magnetic resonance substudy of SUMMIT demonstrated a significant reduction of LV mass and paracardiac adipose tissue volume in treated patients, and that the reduction in LV mass was proportional to weight loss and paralleled by a decrease in left atrial overload (101).

Of note, both in the STEP-HFpEF program and in the SUMMIT trial, very few patients were being treated with SGLT2-Is which would nowadays be standard of care in this patient population. Moreover, data from these trials cannot be applied to non-obese patients with HFpEF and a normal BMI. While it is reasonable that inducing significant weight-loss in an obese patient with HFpEF may enhance quality of life and increase exercise capacity, existing data are not straightforward in supporting a specific cardiac effect on HF beyond weight-loss, as also highlighted by the negative experiences with HFrEF in the FIGHT and LIVE trials. Clinicians should also consider the possible heart rate increase induced by GLP1-RAs, and exercise caution in patients with uncontrolled or previous life-threatening arrhythmias.

### Berberine

Berberine is a quaternary ammonium salt belonging to the group of benzylisochinolone alkaloids. It is found in some plants of the genus Berberis, usually in the roots, rhizomes, stems and bark. It has been used for over 2000 years in Chinese and Indian Ayurvedic medicine as an antidiarrheal, antimicrobial, antineoplastic agent. It has also been used for a long time as an antidiabetic, and published research supports its efficacy in reducing IR (102, 103).

There is also a fair amount of good scientific literature supporting beneficial mechanisms on the cardiovascular system. Experimental studies in a murine “two-hit” HFpEF model demonstrated that berberine determines better tolerance to exercise and improved diastolic function, intervening on the regulation of phospholamban and SERCA2a at the level of the sarcoplasmic reticulum, and protecting from mitochondrial fragmentation (104).

A small, randomized, placebo-controlled trial published in 2003 suggested that the addition of berberine to drug treatment in patients with HFrEF produced a significant improvement in EF, exercise capacity, dyspnea-fatigue index and ventricular arrhythmia (105). Unfortunately, these data have never been replicated in larger and more contemporary populations. In a randomized, double-blind, placebo-controlled study conducted by our group several years ago in 145 patients with metabolic syndrome and left ventricular hypertrophy, 6 months of treatment with berberine determined a significant reduction in LV mass and an improvement in diastolic function parameters, a finding that may support further research in HFpEF patients with IR (57, 58).

## Lifestyle and rehabilitation intervention in HFpEF with IR

Given the importance of obesity and central adiposity in the pathophysiology of HFpEF with IR, much emphasis has always been placed on non-pharmacological interventions aimed at inducing weight loss and reducing physical deconditioning.

Dietary patterns appear to be linked to the development and prognosis of HFpEF (106). Self-reported adherence to the mediterranean diet seemed to correlate with reduced rehospitalization rates in patients with acute HF in the MEDIT-AHF study (107). Lifestyle intervention (i.e., dietary counseling and unsupervised exercise) has proven effective in inducing weight loss and functional improvement (108). Common dietary advice in these patients also includes moderate-to-strict sodium restriction, although the hard evidence on the topic is basically neutral (109). However, the GOURMET-HF trial on recently hospitalized HF patients demonstrated beneficial short-term effects of the DASH diet (a low-sodium, low-calories diet regimen first designed for hypertension) on quality of life and readmissions (110).

Multiple randomized studies have demonstrated the beneficial effects of structured exercise training programs on exercise capacity and quality of life measures (111). These studies are highly heterogeneous in terms of sample size, exercise protocols and time frame, yet the beneficial effects of rehabilitation seem particularly significant in HFpEF patients compared to those with HFrEF and add to the benefit provided by dietary intervention (112). The SECRET trial demonstrated the additive benefits of exercise training and balanced energy-restricted diet on exercise capacity (but not on quality of life) in HFpEF patients (113).

Although no formal specific trials exist on the topic, observational data suggest that bariatric surgery-induced weight loss may favorably impact on readmission rates in HF (114). Of course, surgery is intended for severe obesity where other lifestyle and pharmacological measures have failed, and great care must be taken in preoperative risk assessment in this particularly fragile population.

## Strengths, limitations and future directions

This is a narrative overview approaching an extensive amount of literature, trying to sort out relevant data and connecting them in an organic reading frame. It is beyond the scope of this review to analyze in detail, for example, the statistical intricacies of epidemiological data, or the molecular details of drug pharmacodynamics. We tried to underline aspects where little or no evidence exists, including fields where trials are currently being carried out. The main gap in knowledge still seems to be the missing causal link between a first pathophysiological hit (IR/HI) and a final clinical manifestation (HF); epidemiological data are still equivocal in this regard, with a general scarcity of longitudinal data proving causation. Hence, the very concept of early screening for IR/HI for prevention of HFpEF, albeit intriguing, remains unsupported by hard evidence. Moreover, the possible benefits of drug treatment in subjects with early IR/HI are yet to be demonstrated, both with regard to HF incidence and in terms of general prognostic outlook. Future research will have to address this evidence gaps.

## Conclusions

This review summarizes the existing evidence supporting an association between HFpEF and IR/HI. The literature shows that the latter, through mechanisms that damage cardiovascular structure and function, should be at least considered an important contributory cause of the development and/or worsening of HFpEF. Coherently with these common mechanisms and similar epidemiology, drug treatment and non-pharmacological interventions for IR/T2DM and HFpEF substantially overlap. Whether early identification and treatment of subjects with IR/HI could determine a reduction in the subsequent incidence of HFpEF remains to be ascertained in future research.

## References

[1] Goh LPW Sani SA Sabullah MK Gansau JA. The prevalence of insulin resistance in Malaysia and Indonesia: an updated systematic review and meta-analysis. Medicina. (2022) 58(6):826. 10.3390/medicina58060826 35744089 PMC9227905

[2] Bermudez V Salazar J Martínez MS Chávez-Castillo M Olivar LC Calvo MJ Prevalence and associated factors of insulin resistance in adults from Maracaibo City, Venezuela. Adv Prev Med. (2016) 2016:9405105. 10.1155/2016/9405105 27579182 PMC4989131

[3] Santos L. The impact of nutrition and lifestyle modification on health. Eur J Intern Med. (2022) 97:18–25. 10.1016/j.ejim.2021.09.020 34670680

[4] Savarese G Becher PM Lund LH Seferovic P Rosano GMC Coats AJS. Global burden of heart failure: a comprehensive and updated review of epidemiology. Cardiovasc Res. (2023) 118(17):3272–87. 10.1093/cvr/cvac013 Erratum in: *Cardiovasc Res*. (2023) **119**(6):1453. doi: 10.1093/cvr/cvad026. 35150240

[5] Khan MS Shahid I Bennis A Rakisheva A Metra M Butler J. Global epidemiology of heart failure. Nat Rev Cardiol. (2024) 21(10):717–34. 10.1038/s41569-024-01046-6 38926611

[6] Erqou S Adler AI Challa AA Fonarow GC Echouffo-Tcheugui JB. Insulin resistance and incident heart failure: a meta-analysis. Eur J Heart Fail. (2022) 24(6):1139–41. 10.1002/ejhf.2531 35502564 PMC9262840

[7] Su X Zhao C Zhang X. Association between METS-IR and heart failure: a cross-sectional study. Front Endocrinol. (2024) 15:1416462. 10.3389/fendo.2024.1416462 PMC11249535 39015177

[8] Li X Wang J Niu L Tan Z Ma J He L Prevalence estimates of the insulin resistance and associated prevalence of heart failure among United Status adults. BMC Cardiovasc Disord. (2023) 23(1):294. 10.1186/s12872-023-03294-9 37301866 PMC10257843

[9] Cui DY Zhang C Chen Y Qian GZ Zheng WX Zhang ZH Associations between non-insulin-based insulin resistance indices and heart failure prevalence in overweight/obesity adults without diabetes mellitus: evidence from the NHANES 2001–2018. Lipids Health Dis. (2024) 23(1):123. 10.1186/s12944-024-02114-z 38678275 PMC11055335

[10] Fazio S Mercurio V Tibullo L Fazio V Affuso F. Insulin resistance/hyperinsulinemia: an important cardiovascular risk factor that has long been underestimated. Front Cardiovasc Med. (2024) 11:1380506. 10.3389/fcvm.2024.1380506 38545338 PMC10965550

[11] Fletcher B Lamendola C. Insulin resistance syndrome. J Cardiovasc Nurs. (2004) 19(5):339–45. 10.1097/00005082-200409000-00009 15495894

[12] Janssen JAMJL. Hyperinsulinemia and its pivotal role in aging, obesity, type 2 diabetes, cardiovascular disease and cancer. Int J Mol Sci. (2021) 22(15):7797. 10.3390/ijms22157797 34360563 PMC8345990

[13] Hill MA Yang Y Zhang L Sun Z Jia G Parrish AR Insulin resistance, cardiovascular stiffening and cardiovascular disease. Metab Clin Exp. (2021) 119:154766. 10.1016/j.metabol.2021.154766 33766485

[14] Lebovitz HE. Insulin resistance: definition and consequences. Exp Clin Endocrinol Diabetes. (2001) 109(2):S135–48. 10.1055/s-2001-18576 11460565

[15] Golla MSG Shams P. Heart failure with preserved ejection fraction (HFpEF). In: StatPearls [Internet]. Treasure Island, FL: StatPearls Publishing (2025). 38320083

[16] Freeman AM Acevedo LA Pennings N. Insulin resistance. In: StatPearls [Internet]. Treasure Island, FL: StatPearls Publishing (2025). 29939616

[17] Fazio S Bellavite P Affuso F. Chronically increased levels of circulating insulin secondary to insulin resistance: a silent killer. Biomedicines. (2024) 12(10):2416. 10.3390/biomedicines12102416 39457728 PMC11505545

[18] Kelsey MD Nelson AJ Green JB Granger CB Peterson ED McGuire DK Guidelines for cardiovascular risk reduction in patients with type 2 diabetes: JACC guideline comparison. J Am Coll Cardiol. (2022) 79(18):1849–57. 10.1016/j.jacc.2022.02.046 35512864 PMC8972581

[19] Marx N Federici M Schütt K Müller-Wieland D Ajjan RA Antunes MJ 2023 ESC guidelines for the management of cardiovascular disease in patients with diabetes. Eur Heart J. (2023) 44(39):4043–140. 10.1093/eurheartj/ehad192. Erratum in: *Eur Heart J*. (2023) **44**(48):5060. doi: 10.1093/eurheartj/ehad774. Erratum in: *Eur Heart J*. (2024) **45**(7):518. doi: 10.1093/eurheartj/ehad857. 37622663

[20] Gastaldelli A. Measuring and estimating insulin resistance in clinical and research settings. Obesity (Silver Spring). (2022) 30(8):1549–63. 10.1002/oby.23503 35894085 PMC9542105

[21] de Cassia da Silva C Zambon MP Vasques ACJ Camilo DF de Góes Monteiro Antonio MÂR Geloneze B. The threshold value for identifying insulin resistance (HOMA-IR) in an admixed adolescent population: a hyperglycemic clamp validated study. Arch Endocrinol Metab. (2023) 67(1):119–25. 10.20945/2359-3997000000533 36468919 PMC9983787

[22] Guerrero-Romero F Simental-Mendía LE González-Ortiz M Martínez-Abundis E Ramos-Zavala MG Hernández-González SO The product of triglycerides and glucose, a simple measure of insulin sensitivity. Comparison with the euglycemic-hyperinsulinemic clamp. J Clin Endocrinol Metab. (2010) 95(7):3347–51. 10.1210/jc.2010-0288 20484475

[23] Nishiyama SK Zhao J Wray DW Richardson RS. Vascular function and endothelin-1: tipping the balance between vasodilation and vasocostiction. J Appl Physiol. (2017) 122(2):354–60. 10.1152/japplphysiol.00772.201627 27909229 PMC5338602

[24] Adeva-Andany MM Ameneiros-Rodrìguez E Fernández-Fernández C Dominguez-Montero A Funcasta-Calderón R. Insulin resistance is associated with subclinical vascular disease in humans. World J Diabetes. (2019) 10:63–77. 10.4239/wjd.v10.i2.6328 30788044 PMC6379732

[25] Arauz-Pacheco C Lender D Snell PG Huet B Ramirez LC Breen L Relationship between insulin sensitivity, hyperinsulinemia, and insulin-mediated sympathetic activation in normotensive and hypertensive subjects. Am J Hypertens. (1996) 9(12 Pt1):1172–8. 10.1016/S0895-7061(96)00256-7 8972887

[26] Anderson EA Hoffman RP Balon TW Sinkey CA Mark AL. Hyperinsulinemia produces both sympathetic neural activation and vasodilation in normal humans. J Clin Invest. (1991) 87(6):2246–52. 10.1172/LCI11526031 2040704 PMC296986

[27] Zamolodchikova TS Tolpygo SM Kotov AV. Insulin in the regulation of the renin-angiotensin system: a new perspective on the mechanism of insulin resistance and diabetic complications. Front Endocrinol. (2024) 15:1293221. 10.3389/fendo.2024.1293221 PMC10844507 38323106

[28] Sowers JR. Insulin resistance and hypertension. Am J Physiol Heart Circ Physiol. (2004) 286(5):H1597–602. 10.1152/ajpheart.00026.2004 15072967

[29] Sundström J Lind L Nyström N Zethelius B Andrén B Hales CN Left ventricular concentric remodeling rather than left ventricular hypertrophy is related to the insulin resistance syndrome in elderly men. Circulation. (2000) 101(22):2595–600. 10.1161/01.cir.101.22.2595 10840010

[30] Karaca Ü Schram MT Houben AJ Muris DM Stehouwer CD. Microvascular dysfunction as a link between obesity, insulin resistance and hypertension. Diabetes Res Clin Pract. (2014) 103(3):382–7. 10.1016/j.diabres.2013.12.012 24438874

[31] Naryzhnaya NV Koshelskaya OA Kologrivova IV Kharitonova OA Evtushenko VV Boshchenko AA. Hypertrophy and insulin resistance of epicardial adipose tissue adipocytes: association with the coronary artery disease severity. Biomedicines. (2021) 9(1):64. 10.3390/biomedicines9010064 33440802 PMC7827040

[32] Kalmpourtzidou A Napoli D Vincenti I De Giuseppe A Casali R Tomasinelli PM Epicardial fat and insulin resistance in healthy older adults: a cross-sectional analysis. Geroscience. (2024) 46(2):2123–37. 10.1007/s11357-023-00972-6 37857994 PMC10828363

[33] Iacobellis G Leonetti F. Epicardial adipose tissue and insulin resistance in obese subjects. J Clin Endocrinol Metab. (2005) 90(11):6300–2. 10.1210/jc.2005-1087 16091479

[34] Jia G DeMarco VG Sowers JR. Insulin resistance and hyperinsulinaemia in diabetic cardiomyopathy. Nat Rev Endocrinol. (2016) 12(3):144–53. 10.1038/nrendo.2015.216 26678809 PMC4753054

[35] Packer M. The adipokine hypothesis of heart failure with a preserved ejection fraction: a novel framework to explain pathogenesis and guide treatment. J Am Coll Cardiol. (2025) 86(16):S0735-1097(25)07049-4. 10.1016/j.jacc.2025.06.055 40886173 PMC12766646

[36] Ostrominski JW DeFilippis EM Bansal K Riello RJ 3rd Bozkurt B Heidenreich PA Contemporary American and European guidelines for heart failure management: JACC: heart failure guideline comparison. JACC Heart Fail. (2024) 12(5):810–25. 10.1016/j.jchf.2024.02.020 38583167

[37] Campbell P Rutten FH Lee MM Hawkins NM Petrie MC. Heart failure with preserved ejection fraction: everything the clinician needs to know. Lancet. (2024) 403(10431):1083–92. 10.1016/S0140-6736(23)02756-3 Erratum in: *Lancet*. (2024) **403**(10431):1026. doi: 10.1016/S0140-6736(24)00494-X. 38367642

[38] Ballena-Caicedo J Zuzunaga-Montoya FE Loayza-Castro JA Bustamante-Rodríguez JC Vásquez Romero LEM Tapia-Limonchi R Global prevalence of insulin resistance in the adult population: a systematic review and meta-analysis. Front Endocrinol. (2025) 16:1646258. 10.3389/fendo.2025.1646258 PMC12411212 40917357

[39] Wu C Ke Y Nianogo RA. Trends in hyperinsulinemia and insulin resistance among nondiabetic US adults, NHANES, 1999–2018. J Clin Med. (2025) 14(9):3215. 10.3390/jcm14093215 40364246 PMC12072812

[40] Zhao D Wang L Jiao X Shi C Luo Z Chen Y Trends in prevalence of insulin resistance among nondiabetic/nonprediabetic adolescents, 1999–2020. Pediatr Diabetes. (2025) 2025:9982025. 10.1155/pedi/9982025 40365140 PMC12069839

[41] Kim S Song K Lee M Suh J Chae HW Kim HS Trends in HOMA-IR values among South Korean adolescents from 2007 to 2010 to 2019–2020: a sex-, age-, and weight status-specific analysis. Int J Obes. (2023) 47(9):865–72. 10.1038/s41366-023-01340-2 PMC10439007 37443273

[42] Lin Z Yuan S Li B Guan J He J Song C Insulin-based or non-insulin-based insulin resistance indicators and risk of long-term cardiovascular and all-cause mortality in the general population: a 25-year cohort study. Diabetes Metab. (2024) 50(5):101566. 10.1016/j.diabet.2024.101566 39127168

[43] Zhang X Li J Zheng S Luo Q Zhou C Wang C. Fasting insulin, insulin resistance, and risk of cardiovascular or all-cause mortality in non-diabetic adults: a meta-analysis. Biosci Rep. (2017) 37(5):BSR20170947. 10.1042/BSR20170947 28811358 PMC6448479

[44] Borlaug BA Jensen MD Kitzman DW Lam CSP Obokata M Rider OJ. Obesity and heart failure with preserved ejection fraction: new insights and pathophysiological targets. Cardiovasc Res. (2023) 118(18):3434–50. 10.1093/cvr/cvac120 35880317 PMC10202444

[45] Dunlay SM Roger VL Redfield MM. Epidemiology of heart failure with preserved ejection fraction. Nat Rev Cardiol. (2017) 14(10):591–602. 10.1038/nrcardio.2017.65 28492288

[46] Ahmed B Sultana R Greene MW. Adipose tissue and insulin resistance in obese. Biomed Pharmacother. (2021) 137:111315. 10.1016/j.biopha.2021.111315 33561645

[47] Kurauti MA Soares GM Marmentini C Bronczek GA Branco RCS Boschero AC. Insulin and aging. Vitam Horm. (2021) 115:185–219. 10.1016/bs.vh.2020.12.010 33706949

[48] Cerdas Pérez S. Menopause and diabetes. Climacteric. (2023) 26(3):216–21. 10.1080/13697137.2023.2184252 37011666

[49] Reddy YNV Frantz RP Hemnes AR Hassoun PM Horn E Leopold JA Disentangling the impact of adiposity from insulin resistance in heart failure with preserved ejection fraction. J Am Coll Cardiol. (2025) 85(18):1774–88. 10.1016/j.jacc.2025.03.530 40335254 PMC12890320

[50] Son TK Toan NH Thang N Trong Tuong L Tien H Thuy HA Prediabetes and insulin resistance in a population of patients with heart failure and reduced or preserved ejection fraction but without diabetes, overweight or hypertension. Cardiovasc Diabetol. (2022) 21(1):75. 10.1186/s12933-022-01509-5 35568879 PMC9107647

[51] Li Z Fan X Liu Y Yu L He Y Li L Triglyceride-glucose index is associated with heart failure with preserved ejection fraction in different metabolic states in patients with coronary heart disease. Front Endocrinol. (2024) 15:1447072. 10.3389/fendo.2024.1447072 PMC11570926 39558978

[52] Wamil M Coleman RL Adler AI McMurray JJV Holman RR. Increased risk of incident heart failure and death is associated with insulin resistance in people with newly diagnosed type 2 diabetes: UKPDS 89. Diabetes Care. (2021) 44(8):1877–84. 10.2337/dc21-0429 34162666

[53] Savji N Meijers WC Bartz TM Bhambhani V Cushman M Nayor M The association of obesity and cardiometabolic traits with incident HFpEF and HFrEF. JACC Heart Fail. (2018) 6(8):701–9. 10.1016/j.jchf.2018.05.018 30007554 PMC6076337

[54] Cauwenberghs N Knez J Thijs L Haddad F Vanassche T Yang WY Relation of insulin resistance to longitudinal changes in left ventricular structure and function in a general population. J Am Heart Assoc. (2018) 7(7):e008315. 10.1161/JAHA.117.008315 29574459 PMC5907600

[55] Velagaleti RS Gona P Chuang ML Salton CJ Fox CS Blease SJ Relations of insulin resistance and glycemic abnormalities to cardiovascular magnetic resonance measures of cardiac structure and function: the framingham heart study. Circ Cardiovasc Imaging. (2010) 3(3):257–63. 10.1161/CIRCIMAGING.109.911438 20208015 PMC3057083

[56] Ohya Y Abe I Fujii K Ohmori S Onaka U Kobayashi K Hyperinsulinemia and left ventricular geometry in a work-site population in Japan. Hypertension. (1996) 27(3 Pt 2):729–34. 10.1161/01.hyp.27.3.729 8613232

[57] Carlomagno G Pirozzi C Mercurio V Ruvolo A Fazio S. Effects of a nutraceutical combination on left ventricular remodeling and vasoreactivity in subjects with the metabolic syndrome. Nutr Metab Cardiovasc Dis. (2012) 22(5):e13–4. 10.1016/j.numecd.2011.12.002 22397875

[58] Mercurio V Pucci G Bosso G Fazio V Battista F Iannuzzi A A nutraceutical combination reduces left ventricular mass in subjects with metabolic syndrome and left ventricular hypertrophy: a multicenter, randomized, double-blind, placebo-controlled trial. Clin Nutr. (2020) 39(5):1379–84. 10.1016/j.clnu.2019.06.026 31371114

[59] Ni W Jiang R Xu D Zhu J Chen J Lin Y Association between insulin resistance indices and outcomes in patients with heart failure with preserved ejection fraction. Cardiovasc Diabetol. (2025) 24(1):32. 10.1186/s12933-025-02595-x 39844150 PMC11755915

[60] Shen L Rørth R Cosmi D Kristensen SL Petrie MC Cosmi F Insulin treatment and clinical outcomes in patients with diabetes and heart failure with preserved ejection fraction. Eur J Heart Fail. (2019) 21(8):974–84. 10.1002/ejhf.1535 31271255 PMC7079555

[61] Caturano A Vetrano E Galiero R Sardu C Rinaldi L Russo V Advances in the insulin-heart axis: current therapies and future directions. Int J Mol Sci. (2024) 25(18):10173. 10.3390/ijms251810173 39337658 PMC11432093

[62] Miki T Yuda S Kouzu H Miura T. Diabetic cardiomyopathy: pathophysiology and clinical features. Heart Fail Rev. (2013) 18(2):149–66. 10.1007/s10741-012-9313-3 22453289 PMC3593009

[63] Yang C Liu W Tong Z Lei F Lin L Huang X The relationship between insulin resistance indicated by triglyceride and glucose Index and left ventricular hypertrophy and decreased left ventricular diastolic function with preserved ejection fraction. Diabetes Metab Syndr Obes. (2024) 17:2259–72. 10.2147/DMSO.S454876 38868630 PMC11166847

[64] Matsushita K Harada K Kohno T Nakano H Kitano D Matsuda J Prevalence and clinical characteristics of diabetic cardiomyopathy in patients with acute heart failure. Nutr Metab Cardiovasc Dis. (2024) 34(5):1325–33. 10.1016/j.numecd.2023.12.013 38218713

[65] Segar MW Khan MS Patel KV Butler J Tang WHW Vaduganathan M Prevalence and prognostic implications of diabetes with cardiomyopathy in community-dwelling adults. J Am Coll Cardiol. (2021) 78(16):1587–98. 10.1016/j.jacc.2021.08.020 34649696

[66] Seferović PM Paulus WG. Clinical diabetic cardiomyopathy: a two-faced disease with restrictive and dilated phenotypes. Eur Heart J. (2015) 36(27):1718–27. 10.1093/eurheartj/ehv134 25888006

[67] Jia G Hill MA Sowers JR. Diabetic cardiomyopathy: an update of mechanisms contributing to this clinical entity. Circ Res. (2018) 122(4):624–38. 10.1161/CIRCRESAHA.117.311586 29449364 PMC5819359

[68] Januzzi JL Jr Butler J Del Prato S Ezekowitz JA Ibrahim NE Lam CSP Rationale and design of the aldose reductase inhibition for stabilization of exercise capacity in heart failure trial (ARISE-HF) in patients with high-risk diabetic cardiomyopathy. Am Heart J. (2023) 256:25–36. 10.1016/j.ahj.2022.11.003 36372245

[69] Herman R Kravos NA Jensterle M Janež A Dolžan V. Metformin and insulin resistance: a review of the underlying mechanisms behind changes in GLUT4-mediated glucose transport. Int J Mol Sci. (2022) 23(3):1264. 10.3390/ijms23031264 35163187 PMC8836112

[70] Andersson C Søgaard P Hoffmann S Hansen PR Vaag A Major-Pedersen A Metformin is associated with improved left ventricular diastolic function measured by tissue Doppler imaging in patients with diabetes. Eur J Endocrinol. (2010) 163(4):593–9. 10.1530/EJE-10-0624 20679358

[71] Mohan M Al-Talabany S McKinnie A Mordi IR Singh JSS Gandy SJ A randomized controlled trial of metformin on left ventricular hypertrophy in patients with coronary artery disease without diabetes: the MET-REMODEL trial. Eur Heart J. (2019) 40(41):3409–17. 10.1093/eurheartj/ehz203 30993313 PMC6823615

[72] Schernthaner G Brand K Bailey CJ. Metformin and the heart: update on mechanisms of cardiovascular protection with special reference to comorbid type 2 diabetes and heart failure. Metab Clin Exp. (2022) 130:155160. 10.1016/j.metabol.2022.155160 35143848

[73] King P Peacock I Donnelly R. The UK prospective diabetes study (UKPDS): clinical and therapeutic implications for type 2 diabetes. Br J Clin Pharmacol. (1999) 48(5):643–8. 10.1046/j.1365-2125.1999.00092.x 10594464 PMC2014359

[74] Khan MS Solomon N DeVore AD Sharma A Felker GM Hernandez AF Clinical outcomes with metformin and sulfonylurea therapies among patients with heart failure and diabetes. JACC Heart Fail. (2022) 10(3):198–210. 10.1016/j.jchf.2021.11.001 34895861

[75] Crowley MJ Diamantidis CJ McDuffie JR Cameron CB Stanifer JW Mock CK Clinical outcomes of metformin use in populations with chronic kidney disease, congestive heart failure, or chronic liver disease: a systematic review. Ann Intern Med. (2017) 166(3):191–200. 10.7326/M16-1901 28055049 PMC5293600

[76] Halabi A Sen J Huynh Q Marwick TH. Metformin treatment in heart failure with preserved ejection fraction: a systematic review and meta-regression analysis. Cardiovasc Diabetol. (2020) 19(1):124. 10.1186/s12933-020-01100-w 32758236 PMC7409497

[77] Hosokawa Y Ogawa W. SGLT2 Inhibitors for genetic and acquired insulin resistance: considerations for clinical use. J Diabetes Investig. (2020) 11(6):1431–3. 10.1111/jdi.13309 32469141 PMC7610101

[78] Carluccio E Biagioli P Reboldi G Mengoni A Lauciello R Zuchi C Left ventricular remodeling response to SGLT2 inhibitors in heart failure: an updated meta-analysis of randomized controlled studies. Cardiovasc Diabetol. (2023) 22(1):235. 10.1186/s12933-023-01970-w 37660005 PMC10475184

[79] Tanaka H Hirata KI. Potential impact of SGLT2 inhibitors on left ventricular diastolic function in patients with diabetes mellitus. Heart Fail Rev. (2018) 23(3):439–44. 10.1007/s10741-018-9668-1 29330646

[80] Tadic M Sala C Saeed S Grassi G Mancia G Rottbauer W New antidiabetic therapy and HFpEF: light at the end of tunnel? Heart Fail Rev. (2022) 27(4):1137–46. 10.1007/s10741-021-10106-9 33843015 PMC9197886

[81] Verma S Garg A Yan AT Gupta AK Al-Omran M Sabongui A Effect of empagliflozin on left ventricular mass and diastolic function in individuals with diabetes: an important clue to the EMPA-REG OUTCOME trial? Diabetes Care. (2016) 39(12):e212–3. 10.2337/dc16-1312 27679584

[82] Cohen ND Gutman SJ Briganti EM Taylor AJ. Effects of empagliflozin treatment on cardiac function and structure in patients with type 2 diabetes: a cardiac magnetic resonance study. Intern Med J. (2019) 49(8):1006–10. 10.1111/imj.14260 30784160

[83] Vaduganathan M Docherty KF Claggett BL Jhund PS de Boer RA Hernandez AF SGLT-2 inhibitors in patients with heart failure: a comprehensive meta-analysis of five randomised controlled trials. Lancet. (2022) 400(10354):757–67. 10.1016/S0140-6736(22)01429-5. Erratum in: *Lancet*. (2023) **401**(10371):104. doi: 10.1016/S0140-6736(23)00018-1. 36041474

[84] Pandey AK Bhatt DL Pandey A Marx N Cosentino F Pandey A Mechanisms of benefits of sodium-glucose cotransporter 2 inhibitors in heart failure with preserved ejection fraction. Eur Heart J. (2023) 44(37):3640–51. 10.1093/eurheartj/ehad389 37674356

[85] Gao M Bhatia K Kapoor A Badimon J Pinney SP Mancini DM SGLT2 Inhibitors, functional capacity, and quality of life in patients with heart failure: a systematic review and meta-analysis. JAMA Netw Open. (2024) 7(4):e245135. 10.1001/jamanetworkopen.2024.5135 38573633 PMC11192183

[86] Ussher JR Drucker DJ. Glucagon-like peptide 1 receptor agonists: cardiovascular benefits and mechanisms of action. Nat Rev Cardiol. (2023) 20(7):463–74. 10.1038/s41569-023-00849-3 36977782

[87] Bendotti G Montefusco L Lunati ME Usuelli V Pastore I Lazzaroni E The anti-inflammatory and immunological properties of GLP-1 receptor agonists. Pharmacol Res. (2022) 182:106320. 10.1016/j.phrs.2022.106320 35738455

[88] Mashayekhi M Nian H Mayfield D Devin JK Gamboa JL Yu C Weight loss-independent effect of liraglutide on insulin sensitivity in individuals with obesity and prediabetes. Diabetes. (2024) 73(1):38–50. 10.2337/db23-0356 37874653 PMC10784656

[89] American Diabetes Association Professional Practice Committee. 9. Pharmacologic approaches to glycemic treatment: standards of care in diabetes-2025. Diabetes Care. (2025) 48(1 Suppl 1):S181–206. 10.2337/dc25-S009 39651989 PMC11635045

[90] Margulies KB Hernandez AF Redfield MM Givertz MM Oliveira GH Cole R Effects of liraglutide on clinical stability among patients with advanced heart failure and reduced ejection fraction: a randomized clinical trial. JAMA. (2016) 316(5):500–8. 10.1001/jama.2016.10260 27483064 PMC5021525

[91] Jorsal A Kistorp C Holmager P Tougaard RS Nielsen R Hänselmann A Effect of liraglutide, a glucagon-like peptide-1 analogue, on left ventricular function in stable chronic heart failure patients with and without diabetes (LIVE)-a multicentre, double-blind, randomised, placebo-controlled trial. Eur J Heart Fail. (2017) 19(1):69–77. 10.1002/ejhf.657 27790809

[92] Neves JS Vasques-Nóvoa F Borges-Canha M Leite AR Sharma A Carvalho D Risk of adverse events with liraglutide in heart failure with reduced ejection fraction: a *post hoc* analysis of the FIGHT trial. Diabetes Obes Metab. (2023) 25(1):189–97. 10.1111/dom.14862 36082522 PMC9742170

[93] Kosiborod MN Deanfield J Pratley R Borlaug BA Butler J Davies MJ Semaglutide versus placebo in patients with heart failure and mildly reduced or preserved ejection fraction: a pooled analysis of the SELECT, FLOW, STEP-HFpEF, and STEP-HFpEF DM randomised trials. Lancet. (2024) 404(10456):949–61. 10.1016/S0140-6736(24)01643-X 39222642

[94] Kosiborod MN Petrie MC Borlaug BA Butler J Davies MJ Hovingh GK Semaglutide in patients with obesity-related heart failure and type 2 diabetes. N Engl J Med. (2024) 390(15):1394–407. 10.1056/NEJMoa2313917 38587233

[95] Verma S Petrie MC Borlaug BA Butler J Davies MJ Kitzman DW Inflammation in obesity-related HFpEF: the STEP-HFpEF program. J Am Coll Cardiol. (2024) 84(17):1646–62. 10.1016/j.jacc.2024.08.028 39217564

[96] Solomon SD Ostrominski JW Wang X Shah SJ Borlaug BA Butler J Effect of semaglutide on cardiac structure and function in patients with obesity-related heart failure. J Am Coll Cardiol. (2024) 84(17):1587–602. 10.1016/j.jacc.2024.08.021 Erratum in: *J Am Coll Cardiol*. (2025) **85**(9):967–8. doi: 10.1016/j.jacc.2025.01.010. 39217567

[97] Shah SJ Sharma K Borlaug BA Butler J Davies M Kitzman DW Semaglutide and diuretic use in obesity-related heart failure with preserved ejection fraction: a pooled analysis of the STEP-HFpEF and STEP-HFpEF-DM trials. Eur Heart J. (2024) 45(35):3254–69. 10.1093/eurheartj/ehae322 38739118 PMC11400859

[98] Pandey A Kitzman DW Chinnakondepalli KM Patel S Borlaug BA Butler J Frailty and effects of semaglutide in obesity-related HFpEF: findings from the STEP-HFpEF program. JACC Heart Fail. (2025) 13(10):102610. 10.1016/j.jchf.2025.102610 40956259

[99] Frias JP De Block C Brown K Wang H Thomas MK Zeytinoglu M Tirzepatide improved markers of islet cell function and insulin sensitivity in people with T2D (SURPASS-2). J Clin Endocrinol Metab. (2024) 109(7):1745–53. 10.1210/clinem/dgae038 38252888 PMC11180500

[100] Packer M Zile MR Kramer CM Baum SJ Litwin SE Menon V Tirzepatide for heart failure with preserved ejection fraction and obesity. N Engl J Med. (2025) 392(5):427–37. 10.1056/NEJMoa2410027 39555826

[101] Kramer CM Borlaug BA Zile MR Ruff D DiMaria JM Menon V Tirzepatide reduces LV mass and paracardiac adipose tissue in obesity-related heart failure: SUMMIT CMR substudy. J Am Coll Cardiol. (2025) 85(7):699–706. 10.1016/j.jacc.2024.11.001 39566869

[102] Utami AR Maksum IP Deawati Y. Berberine and its study as an antidiabetic compound. Biology. (2023) 12(7):973. 10.3390/biology12070973 37508403 PMC10376565

[103] Cao C Su M. Effects of berberine on glucose-lipid metabolism, inflammatory factors and insulin resistance in patients with metabolic syndrome. Exp Ther Med. (2019) 17(4):3009–14. 10.3892/etm.2019.7295 30936971 PMC6434235

[104] Abudureyimu M Yang M Wang X Luo X Ge J Peng H Berberine alleviates myocardial diastolic dysfunction by modulating Drp1-mediated mitochondrial fission and Ca^2+^ homeostasis in a murine model of HFpEF. Front Med. (2023) 17(6):1219–35. 10.1007/s11684-023-0983-0 37656418

[105] Zeng XH Zeng XJ Li YY. Efficacy and safety of berberine for congestive heart failure secondary to ischemic or idiopathic dilated cardiomyopathy. Am J Cardiol. (2003) 92(2):173–6. 10.1016/s0002-9149(03)00533-2 12860219

[106] Billingsley HE Carbone S Driggin E Kitzman DW Hummel SL. Dietary interventions in heart failure with preserved ejection fraction: a scoping review. JACC Adv. (2024) 4(1):101465. 10.1016/j.jacadv.2024.101465 39801812 PMC11719370

[107] Miró Ò Estruch R Martín-Sánchez FJ Gil V Jacob J Herrero-Puente P Adherence to Mediterranean diet and all-cause mortality after an episode of acute heart failure: results of the MEDIT-AHF study. JACC Heart Fail. (2018) 6(1):52–62. 10.1016/j.jchf.2017.09.020 29226819

[108] Lee VYJ Houston L Perkovic A Barraclough JY Sweeting A Yu J The effect of weight loss through lifestyle interventions in patients with heart failure with preserved ejection fraction-A systematic review and meta-analysis of randomised controlled trials. Heart Lung Circ. (2024) 33(2):197–208. 10.1016/j.hlc.2023.11.022 38320881

[109] Colin-Ramirez E Sepehrvand N Rathwell S Ross H Escobedo J Macdonald P Sodium restriction in patients with heart failure: a systematic review and meta-analysis of randomized clinical trials. Circ Heart Fail. (2023) 16(1):e009879. 10.1161/CIRCHEARTFAILURE.122.009879 36373551

[110] Hummel SL Karmally W Gillespie BW Helmke S Teruya S Wells J Home-delivered meals postdischarge from heart failure hospitalization. Circ Heart Fail. (2018) 11(8):e004886. 10.1161/CIRCHEARTFAILURE.117.004886 30354562 PMC6205816

[111] Mirzai S Sandesara U Haykowsky MJ Brubaker PH Kitzman DW Peters AE. Aerobic, resistance, and specialized exercise training in heart failure with preserved ejection fraction: a state-of-the-art review. Heart Fail Rev. (2025) 30(5):1015–34. 10.1007/s10741-025-10526-x 40372567 PMC12296771

[112] Mentz RJ Whellan DJ Reeves GR Pastva AM Duncan P Upadhya B Rehabilitation intervention in older patients with acute heart failure with preserved versus reduced ejection fraction. JACC Heart Fail. (2021) 9(10):747–57. 10.1016/j.jchf.2021.05.007 34246602 PMC8487922

[113] Kitzman DW Brubaker P Morgan T Haykowsky M Hundley G Kraus WE Effect of caloric restriction or aerobic exercise training on peak oxygen consumption and quality of life in obese older patients with heart failure with preserved ejection fraction: a randomized clinical trial. JAMA. (2016) 315(1):36–46. 10.1001/jama.2015.17346 26746456 PMC4787295

[114] Berger S Meyre P Blum S Aeschbacher S Ruegg M Briel M Bariatric surgery among patients with heart failure: a systematic review and meta-analysis. Open Heart. (2018) 5(2):e000910. 10.1136/openhrt-2018-000910 30613414 PMC6307626

